# Structured Reporting in the Characterization of Renal Cysts by Contrast-Enhanced Ultrasound (CEUS) Using the Bosniak Classification System—Improvement of Report Quality and Interdisciplinary Communication

**DOI:** 10.3390/diagnostics11020313

**Published:** 2021-02-15

**Authors:** Moritz L. Schnitzer, Laura Sabel, Vincent Schwarze, Constantin Marschner, Matthias F. Froelich, Philipp Nuhn, Yannick Falck, Maria-Magdalena Nuhn, Saif Afat, Michael Staehler, Johannes Rückel, Dirk-André Clevert, Johannes Rübenthaler, Thomas Geyer

**Affiliations:** 1 Department of Radiology, University Hospital, LMU Munich, Marchioninistr. 15, 81377 Munich, Germany; moritz.schnitzer14@gmail.com (M.L.S.); lauranina.sabel@gmail.com (L.S.); Vincent.schwarze@med.uni-muenchen.de (V.S.); constantin.marschner@med.uni-muenchen.de (C.M.); Yannick_falck@web.de (Y.F.); Magdalena.nuhn@gmail.com (M.-M.N.); Johannes.rueckel@med.uni-muenchen.de (J.R.); dirk.clevert@med.uni-muenchen.de (D.-A.C.); Johannes.ruebenthaler@med.uni-muenchen.de (J.R.); 2 Department of Radiology and Nuclear Medicine, University Medical Centre Mannheim, Theodor-Kutzer-Ufer 1-3, 68167 Mannheim, Germany; Matthias.froelich@medma.uni-heidelberg.de; 3 Department of Urology, University Medical Centre Mannheim, University of Heidelberg, Theodor-Kutzer-Ufer 1-3, 68167, Mannheim, Germany; nuhn.philipp@gmail.com; 4 Department for Diagnostic and Interventional Radiology, Eberhard Karls University Tuebingen, University Hospital Tuebingen, Hoppe-Seyler-Str. 3, 72076 Tuebingen, Germany; saif.afat@med.uni-tuebingen.de; 5 Department of Urology, University Hospital, LMU Munich, Marchioninistr. 15, 81377 Munich, Germany; Michael.staehler@med.uni-muenchen.de

**Keywords:** structured reporting, CEUS, contrast-enhanced ultrasound, Bosniak classification, renal lesions

## Abstract

Background: This study aims to evaluate the potential benefits of structured reporting (SR) compared to conventional free-text reporting (FTR) in contrast-enhanced ultrasound (CEUS) of cystic renal lesions, based on the Bosniak classification. Methods: Fifty patients with cystic renal lesions who underwent CEUS were included in this single-center study. FTR created in clinical routine were compared to SR retrospectively generated by using a structured reporting template. Two experienced urologists evaluated the reports regarding integrity, effort for information extraction, linguistic quality, and overall quality. Results: The required information could easily be extracted by the reviewers in 100% of SR vs. 82% of FTR (*p* < 0.001). The reviewers trusted the information given by SR significantly more with a mean of 5.99 vs. 5.52 for FTR (*p* < 0.001). SR significantly improved the linguistic quality (6.0 for SR vs. 5.68 for FTR (*p* < 0.001)) and the overall report quality (5.98 for SR vs. 5.58 for FTR (*p* < 0.001)). Conclusions: SR significantly increases the quality of radiologic reports in CEUS examinations of cystic renal lesions compared to conventional FTR and represents a promising approach to facilitate interdisciplinary communication in the future.

## 1. Introduction

In recent years, the incidental detection of cystic renal lesions has increased due to the more widespread use of cross-sectional imaging techniques [1]. At the age of 50 years or older, cystic renal lesions appear in more than half of the patients [2]. The differentiation between simple benign and complicated cystic renal lesions remains a major challenge in modern imaging. About 6% of all asymptomatic renal lesions reveal as cystic renal-cell carcinomas (RCC) [3]. Complicating characteristics including contrast-enhancement, lesional wall thickening, solid components, or intracystic septa can be visualized in up to 10% of incidentally detected renal lesions. In 1986, the Bosniak classification system was introduced and modified in the following years in order to stratify the risk of malignancy of renal cysts [4]. According to the classification, cystic renal lesions can be divided into subtypes with a low risk of malignancy (Bosniak I-II, nearly 0%) and subtypes with elevated rates of malignancy: Bosniak IIF (“F” = follow-up), Bosniak III, and Bosniak IV with a risk of malignancy of approximately 5%, 50%, and 100%, respectively [5,6,7].

Contrast-enhanced ultrasound (CEUS) is an increasingly used imaging modality with high diagnostic accuracy in characterizing unclear renal lesions with higher temporal and spatial resolution than computed tomography (CT) and magnetic resonance imaging (MRI) [8,9]. Previous studies had shown that CEUS allows for reliably stratifying renal lesions into Bosniak subtypes [10,11]. Of note, the use of CEUS rather than cross-sectional imaging offers several benefits including cost-effectiveness, accessibility, and its excellent safety profile [12]. CEUS can be used regardless of metallic foreign bodies and the patient’s renal or thyroid function, and may be safely applied even in children and pregnant women [13,14,15].

Over the past years, several leading radiological societies recommended using structured reporting (SR) rather than conventional free-text reporting (FTR) in order to standardize radiologic reporting and improve report quality and interdisciplinary communication [16,17,18,19]. Previous studies on the impact of SR using various diagnostic imaging modalities indicated that SR can lead to higher satisfaction levels of referring physicians by improving the integrity, readability, and overall quality of radiologic reports [20,21,22,23,24]. However, few contradictory studies pointed out potential disadvantages of SR including possible oversimplification or reduced diagnostic accuracy of radiologic reports [16,25,26].

This retrospective study aimed to evaluate the use of SR rather than FTR in CEUS examinations of cystic renal lesions and its impact on report integrity, effort for information extraction, linguistic quality, and overall report quality to improve interdisciplinary communication in the future.

## 2. Materials and Methods

### 2.1. Study Design

**Figure 1:**
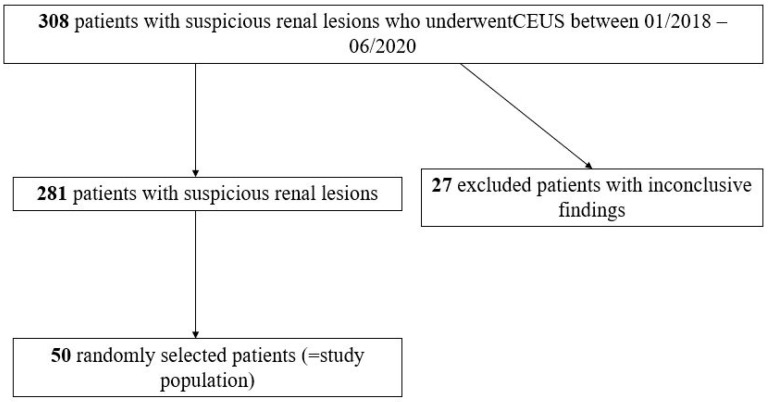
Flowchart illustrating the included patients with suspicious renal lesions who underwent contrast-enhanced ultrasound (CEUS) for further characterization and excluded patients.

This retrospective single-center study was approved by the institutional review board (Ethics Committee, Medical Faculty, Ludwig-Maximilians-University Munich; 17-087; date of approval: 14 March 2017). Informed consent was waived. All authors followed the ethical guidelines for publication in *Diagnostics*. All data were collected according to the principles expressed in the Declaration of Helsinki/Edinburgh 2002. We identified patients with suspicious renal lesions who underwent CEUS at our University Hospital by retrospectively searching our institutional radiology information system. Fifty randomly selected patients with unclear renal lesions who underwent CEUS for further characterization were included in this study (Figure 1). Detailed clinical information provided by the referring physicians were available in all cases.

### 2.2. Sample Size Calculation

The sample size of this study was calculated based on previous studies evaluating the impact of SR in radiology [25,26,27]. The number of reports was determined based on the anticipated effect size when comparing the percentage of reports with completeness of at least 80%. We estimated, as previously described, that 55% of conventional FTR would be rated as high or very high (>80%). In addition, we estimated that up to 70% of SR would be rated as high or very high. Using these estimations, the required sample size was n = 82 (41 FTR, 41 SR). The power was set at 80% and the level of significance at *p* = 0.05. We increased our sample size to n = 100 in order to overcome a possible overestimation of the effect size.

### 2.3. Image Acquisition and Generation of Free-Text Reports

**Figure 2:**
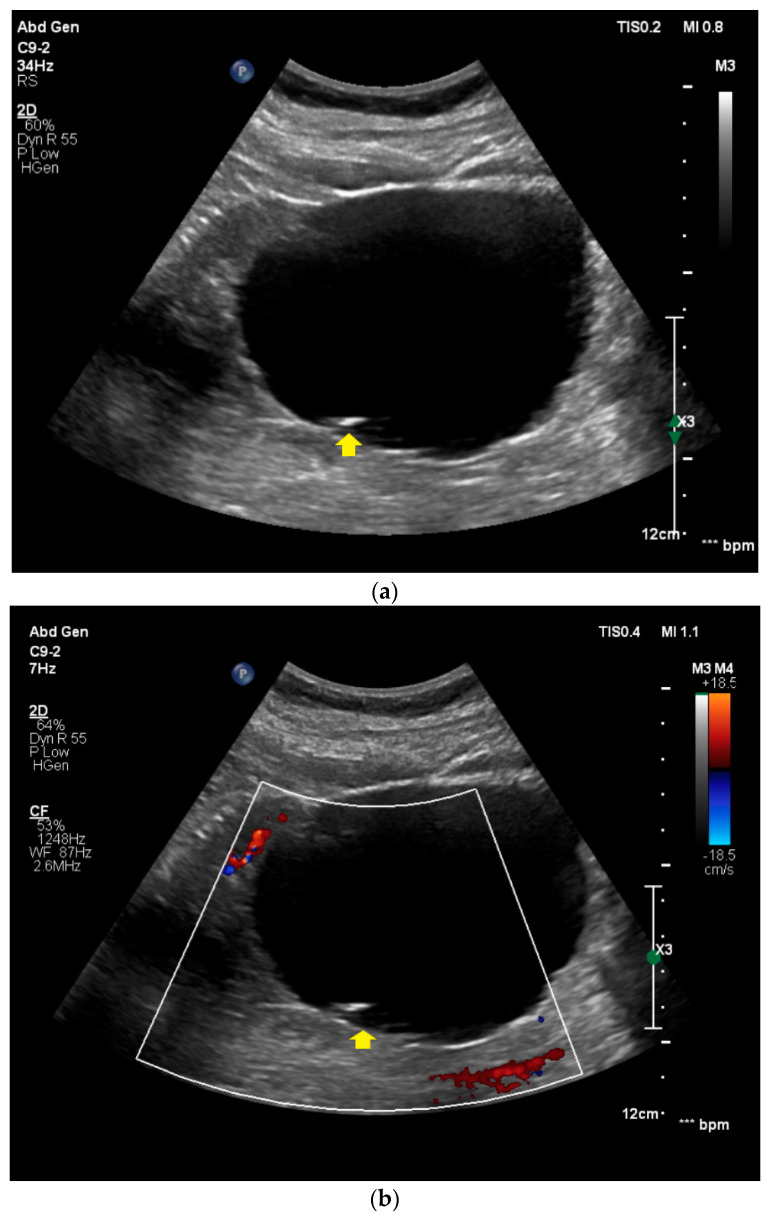

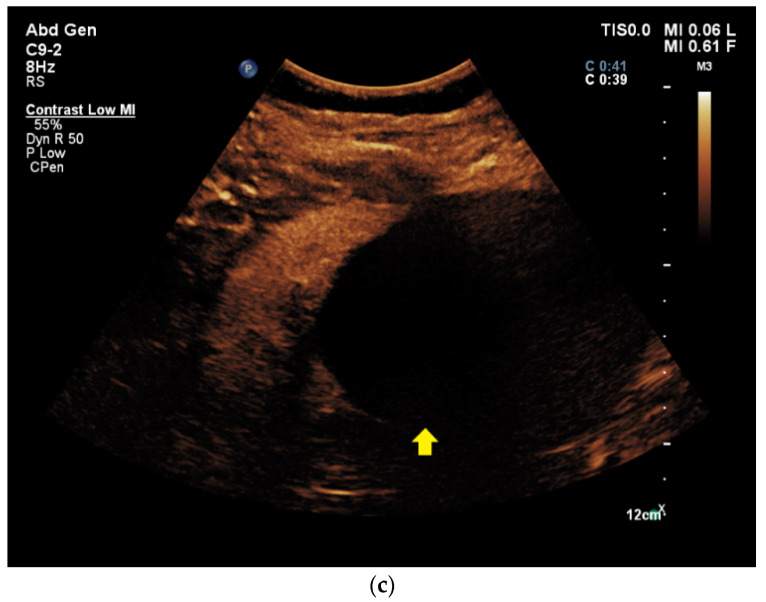
Contrast-enhanced ultrasound of a Bosniak II renal cyst. (**a**) In the native B-mode, a hypoechoic, homogeneous lesion with thin peripheral septa (yellow arrow) is detected in the right kidney. (**b**) The lesion shows no hypervascularization in Color Doppler (yellow arrow). (**c**) After the intravenous application of SonoVue^®^, no contrast-enhancement of the lesion can be visualized (yellow arrow).

All the CEUS examinations were conducted by a single consultant radiologist with over 15 years of professional experience (EFSUMB Level 3) and included the native B-mode ultrasound, Color Doppler, and CEUS (Figure 2). High-end ultrasound devices with adequate CEUS protocols were used with all the patients (Samsung RS80 and RS 85, Seoul, Korea; Philips EPIQ 7, Seattle, WA, USA). A low mechanical index (<0.2) was used in all the examinations to prevent an early destruction of microbubbles. For CEUS, 1.6 to 2.4 mL of the second-generation blood pool contrast agent SonoVue^®^ (Bracco Imaging, Milan, Italy) were intravenously applied followed by 5–10 mL of sterile 0.9% sodium chloride. No adverse side effects after the injection of SonoVue^®^ were registered. Image quality was sufficient in all the examinations and allowed for adequately assessing sonomorphology of the renal lesions of interest. The size, shape, and echogenicity of the lesions were assessed by the B-mode ultrasound. Color Doppler was used to evaluate the lesion vascularization. In CEUS, the lesions were evaluated during the cortical phase (8–35 s after i.v. administration of SonoVue^®^), the corticomedullary phase (36–120 s after i.v. administration of SonoVue^®^), and the late phase (>120 s after i.v. administration of SonoVue^®^). The consultant radiologist who performed the CEUS examinations created all the conventional free-text reports (FTR) which were used for this study in clinical routine immediately after the examinations. All the FTR were created using a speech recognition software system without using predefined text modules (Philips SpeechMagic 6.1, Build 543 SP1 (7/2007), Philips Speech Recognition Systems GmbH, Vienna, Austria). For this study, the FTR were retrospectively taken from our institutional radiology information system.

### 2.4. Generation of Structured Reports

**Figure 3:**
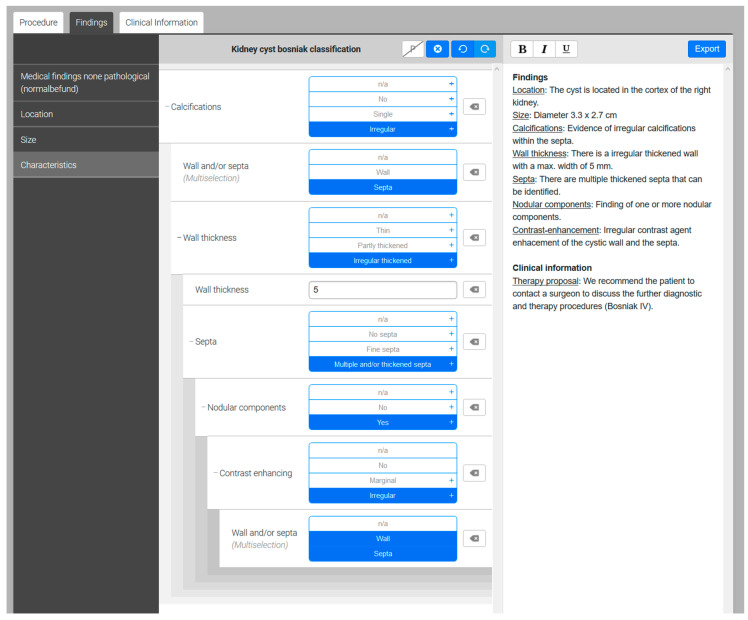
Screenshot of the clickable decision-tree provided by the structured reporting software. The template provides several clickable items and subitems on the left side. By clicking the appropriate items, semantic sentences including Bosniak classification and appropriate therapy proposals are generated by the software on the right side.

All the SR were retrospectively created by a board-certified radiologist from our University Hospital (over 5 years of professional experience) by analyzing the acquired CEUS images in our radiology information system, the clinical patient information provided by the referring physician, and the referring physician’s key questions. An online-based software offering dedicated structured reporting templates was used for generating SR (Smart Reporting GmbH, http://www.smart-reporting.com, accessed 02/02/2021). The structured template with clickable decision trees which was used for our study was created by an experienced board-certified radiologist from our University Hospital based on the Bosniak classification system [3]. By clicking adequate items and subitems in the decision tree, the online software generates semantic sentences based on predefined text phases which can be exported after finishing the SR (Figure 3). No manual editing of the report by the radiologist is necessary.

### 2.5. Evaluation of CEUS Reports

**Figure 4:**
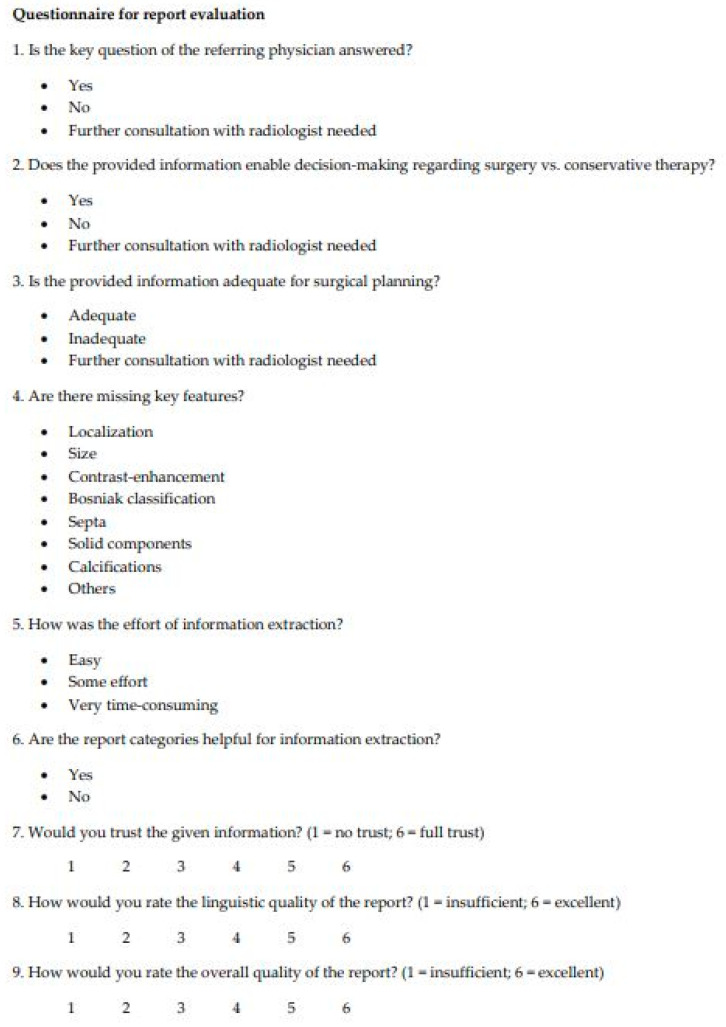
Questionnaire used for the evaluation of free-text reports and structured reports by the two reviewers.

For assessing the quality of FTR and SR and their impact on further patient management, all the reports were evaluated by two board-certified urologists using a questionnaire (Figure 4). The questionnaire was developed based on previous studies which evaluated the impact of SR [22,23]. The first questions covered the following topics: Answering the referring physician’s key question, facilitating clinical decision making, report integrity, and effort for information extraction. In addition, the clinician’s trust in the reports, the linguistic quality, and the overall report quality were evaluated using a 6-point Likert scale (1 = insufficient; 6 = excellent). After anonymizing and randomizing all 100 reports (50 SR + 50 FTR), the reports were read by the two experienced urologists. The questionnaires were answered by the urologists immediately after reading the respective reports.

### 2.6. Statistical Analysis

The statistical calculations were performed using a proprietary statistical software (IBM SPSS Statistics Version 25, Armonk, New York, NY, USA). The McNemar test was used to compare binomial data and the Wilcoxon signed-rank test was used to compare paired data. Cohen’s kappa test was used to evaluate the agreement of both reviewers. The significance level was set at alpha = 0.05.

## 3. Results

We included 50 patients with unclear renal cysts who underwent CEUS at our University Hospital for further characterization. Both reviewers answered 50 questionnaires on FTR and 50 questionnaires on SR, respectively (100 FTR questionnaires and 100 SR questionnaires in total). The reviewers’ response rates were 100%. The agreement between both reviewers was substantial (Cohen’s kappa of 0.665). The results are presented as percentages or as the mean ± standard deviation and 95% confidence interval (CI).

All the SR (100%) answered the key question of the referring physicians vs. 82% of FTR (*p* < 0.001). All the SR (100%) provided sufficient information to enable adequate clinical decision-making between surgery vs. conservative therapy compared to 87% of FTR (*p* < 0.001). The given information was sufficient for surgical planning in 99% of SR vs. 83% of FTR *p* < 0.001). Further consultation with radiologists before surgery was necessary in 1% of SR vs. 13% of FTR (*p* = 0.002).

In addition, 98% of SR were complete without any missing key features, whereas at least one key feature was missing in 26% of FTR (*p* < 0.001). Only one key feature was missing in 2% of SR vs. two or more missing key features in 15% of FTR.

The required information could be easily extracted by the reviewers in 100% of SR vs. 82% of FTR (*p* < 0.001). Among the FTR, “some effort” was needed for information extraction in 17% of the cases, and the process was rated as “very time-consuming” in 1%.

In 100% of SR, the report structure was rated as helpful compared to 97% of FTR.

**Figure 5:**
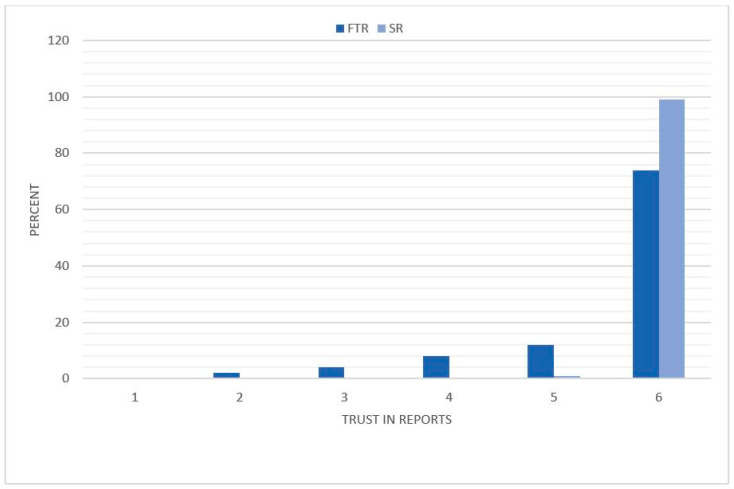
Distribution of the referring physicians’ trust in the report. The reviewers rated the reports based on a Likert scale with a range from 1 to 6 (1 = insufficient, 6 = excellent). The degree of the referring physician’s trust is illustrated on the *x*-axis and the percentage distribution on the *y*-axis. FTR = free text reports; SR = structured reports.

The reviewers trusted the information given by SR significantly more with a mean of 5.99 ± 0.10 (CI: 5.97–6.01) on the Likert scale vs. 5.52 ± 0.95 (CI: 5.33–5.71) for FTR (*p* < 0.001) (Figure 5).

**Figure 6:**
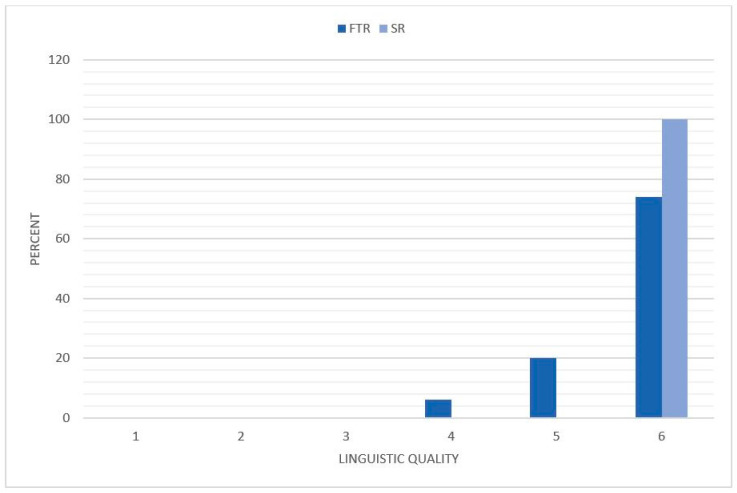
Distribution of the linguistic quality of the reports. The reviewers rated the reports based on a Likert scale with a range from 1 to 6 (1 = insufficient, 6 = excellent). The degree of the linguistic quality is illustrated on the *x*-axis and the percentage distribution on the *y*-axis. FTR = free text reports; SR = structured reports.

The SR received significantly higher ratings regarding the linguistic quality of the reports with a mean of 6.0 ± 0 compared to 5.68 ± 0.58 (CI: 5.56–5.80) for FTR (*p* < 0.001) (Figure 6). Whereas, all the SR received a rating of 6 on the Likert scale (100%), the linguistic quality was considered lower in 26% of FTR with several ratings of 5 (20%) and 4 (6%).

**Figure 7:**
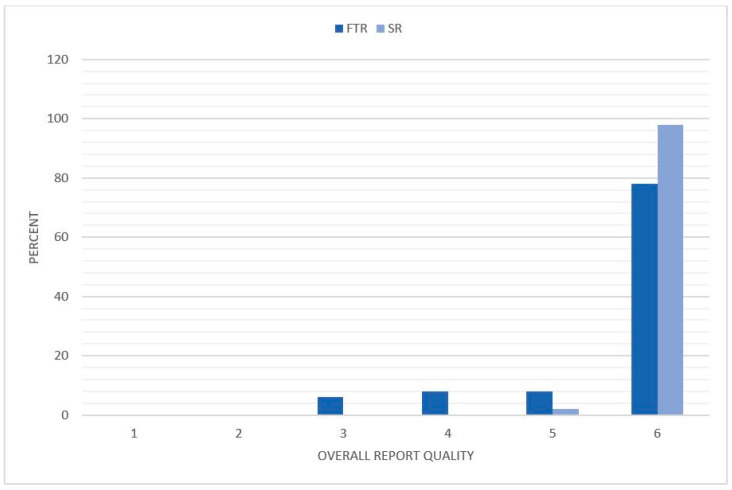
Distribution of the overall report quality. The reviewers rated the reports based on a Likert scale with a range from 1 to 6 (1 = insufficient, 6 = excellent). The degree of the overall report quality is illustrated on the *x*-axis and the percentage distribution on the *y*-axis. FTR = free text reports; SR = structured reports.

The overall report quality was significantly improved by using SR compared to FTR. Whereas, FTR were rated with 5.58 ± 0.88 (CI: 5.41–5.75) and the mean rating for SR was 5.98 ± 0.14 (CI: 5.95–6.01) (*p* < 0.001) (Figure 7). The overall report quality was rated 5 (2%) or 6 (98%) on the Likert scale for all the SR. FTR received a rating of 5 in 8% of the cases, and also received lower ratings of 4 (8%) and 3 (6%).

## 4. Discussion

The contrast-enhanced ultrasound is an increasingly utilized imaging modality with a high diagnostic accuracy for characterizing renal lesions [28,29,30]. Previous studies demonstrated that CEUS is more sensitive in detecting the contrast-enhancement of cystic renal lesions than contrast-enhanced CT and MRI [31,32]. Furthermore, using CEUS rather than cross-sectional imaging for assessing renal lesions brings several benefits such as lower costs, fast availability, and an excellent safety profile.

Since its introduction, the Bosniak classification system has helped radiologists stratify cystic renal lesions according to their risk of malignancy. While the Bosniak classification was originally developed for contrast-enhanced CT, the European Federation of Societies for Ultrasound in Medicine and Biology (EFSUMB) proposed a CEUS-adapted Bosniak categorization system in 2020 in order to overcome differences in imaging specifics and improve the diagnostic accuracy of CEUS in characterizing cystic renal lesions [33]. A recent study evaluating the impact of the updated Bosniak classification in MRI demonstrated higher interobserver agreement and higher diagnostic accuracy when using the updated version [34]. All in all, the Bosniak classification system is a useful tool to make radiologic reporting more standardized and structured, thus facilitating further patient management.

In recent years, various studies previously evaluated the impact of using SR rather than conventional FTR on the quality of radiologic reports, on interdisciplinary communication between radiologists and referring physicians, and on patient management. Several authors proposed SR as a potential tool to facilitate clinical decision-making and improve therapeutic management. However, most studies on the impact of SR focused on cross-sectional imaging modalities (CT, MRI) or X-ray. While Ernst et al. evaluated the use of SR in the native head and neck ultrasound [20], yet, the value of SR in the abdominal ultrasound or in CEUS has not been described. Therefore, our study aimed to identify the potential benefits of SR compared to FTR in CEUS examinations of cystic renal lesions.

Our results demonstrate that using SR rather than FTR significantly increases the integrity, the linguistic quality, and the overall report quality of radiologic reports. Furthermore, the urologists who reviewed the reports trusted the information given by SR significantly more. These findings support several previous studies on the value of SR compared to FTR [21,22,23,24,26,35]. The reviewers perceived that the effort for extracting information from the SR is significantly less time-consuming than FTR. This is in line with previous publications which described a higher effort for information extraction when using FTR [22,28,36].

In our study, relevant features were missing significantly more in FTR compared to SR. While creating SR, the reporting radiologist is automatically reminded of every single key feature by following the hierarchy of the clickable decision-tree in the structured template. Especially radiologists in the beginning of their training or medical students may benefit significantly from this systematic reporting approach, which was indicated by previous studies [28,37,38]. On the contrary, more experienced radiologists who only used FTR in their learning process may be initially faced with difficulties such as prolonged reporting times when using SR. Whereas, some authors described reduced reporting times by using SR and contradictory publications described improved time-efficiency when using a conventional FTR [24,26,29].

Several authors described further possible downsides of using SR rather than FTR. Mentioned concerns include possible oversimplification of radiologic reports, distraction of the reporting radiologists, decreased report quality, and reduced diagnostic accuracy [16,25,26]. In 2009, Johnson et al. found no significant difference between FTR and SR regarding the report integrity and diagnostic accuracy in a study on MRI in stroke patients [39]. Therefore, SR should not be considered as preferable over FTR in general. Its value and its impact on the report quality and interdisciplinary communication should be separately assessed for each imaging modality and software template.

In the near future, radiology will undergo substantial changes along with widespread use of artificial intelligence and radiomics which will provide software-based decision support systems and automated reporting tools in order to make radiologic reporting and radiologic research more efficient [36,40]. Whereas, using conventional FTR usually results in a broad range of individual reporting structures, SR may represent a valuable future approach for facilitating automated information extraction by a dedicated software and thus, advancing the implementation of artificial intelligence in radiology [41]. Recent publications already demonstrated the high potential of a widespread use of SR in radiology as research could be facilitated substantially and the machine learning software could be implemented more easily [37]. In this context, Bosmans et al. described SR as a “fusion reactor hungry for fuel” [38]. Up to now, radiologic reports of CEUS examinations are almost exclusively created by using narrative FTR in routine clinical practice. Our study is the first to show that SR can significantly improve the quality of CEUS reports compared to FTR and thus, facilitate further patient management. Considering the high potential of SR for facilitating the implementation of artificial intelligence software in radiology, our study is an important step to increase acceptance and utilization of SR in ultrasound examinations.

Our study has several limitations. First of all, all the FTR were generated by only one consultant radiologist. The significance of our results regarding the linguistic quality of the reports and the effort of information extraction may be reduced due to the small number of reporting radiologists. The physicians who evaluated the reports might have preferred the linguistic quality of SR due to the individual linguistic reporting style of the experienced radiologist who created the FTR. However, our study also demonstrated that SR significantly improved several aspects which are independent of the individual linguistic reporting style, such as the number of missing key features as well as the provided information for adequate clinical decision-making and surgical planning. The circumstance that FTR and SR were not created by the same radiologist due to the retrospective study setting represents another limitation. However, we aimed to reduce this limitation by only using FTR and SR which were created by highly experienced board-certified radiologists. The applied methods are similar to those of previous works investigating the impact of SR compared to FTR with only one or two radiologists creating the radiologic reports of one category (SR or FTR, respectively) [20,21,36,41]. Furthermore, our retrospective study could not evaluate if using SR rather than FTR has the potential to reduce the reporting times. Moreover, only two urologists evaluated the reports in our study. In order to reduce the limiting impact of this circumstance, both reviewers were experienced consultant urologists with high expertise in the diagnosis and treatment of renal lesions who independently and voluntarily reviewed the questionnaires in our study. The value of SR compared to FTR needs to be further evaluated in prospective studies with a variety of both reporting radiologists and reviewing physicians. A prospective study setting would also allow for assessing the impact of using SR on the reporting times. While information extraction was considered easier from SR than from FTR, the time the urologists needed for interpreting the reports was not actually measured. However, a further consultation with the radiologist was required in significantly more cases of FTR compared to SR, thus indicating that using SR is actually more time-saving. Furethermore, it should be mentioned that all the SR in our study were generated by using an online software template with clickable decision-trees. Therefore, our study cannot be generalized to all forms of SR since other SR approaches are not templated-based decision-trees, but, for example, only offer a hierarchical reporting structure combined with the use of free-text elements. Of note, this study focused on only one imaging modality for assessing the impact of SR compared to FTR in the characterization of renal cysts. As results from ultrasound examinations rely on the subjective assessment of one examiner, further studies should evaluate SR in both the CT and ultrasound simultaneously in order to further validate the results from our study.

In conclusion, our study shows the beneficial use of SR rather than conventional FTR results with increased integrity, readability, and overall quality of radiologic reports in CEUS examinations of cystic renal lesions, thereby facilitating interdisciplinary communication between radiologists and referring physicians.
